# Synthesis, Spectroscopic Characterization, and Biological Evaluation Studies of 5-Bromo-3-(((hydroxy-2-methylquinolin-7-yl)methylene)hydrazono)indolin-2-one and Its Metal (II) Complexes

**DOI:** 10.1155/2014/483282

**Published:** 2014-10-12

**Authors:** Kuruba Siddappa, Nabiya Sultana Mayana

**Affiliations:** Department of Studies and Research in Chemistry, Gulbarga University, Gulbarga, Karnataka 585106, India

## Abstract

The Schiff base ligand 5-bromo-3-(((8-hydroxy-2-methylquinolin-7-yl)methylene)hydrazono)indolin-2-one (BHMQMHI) was prepared *via* condensation of 5-bromo-3-hydrazonoindolin-2-one and 7-formyl-8-hydroxy-2-methylquinoline and its Co(II), Ni(II), Cu(II), Zn(II), Cd(II), and Hg(II) complexes have been synthesized and characterized by elemental analysis, conductance data, magnetic susceptibility measurements, IR, UV-Vis, mass spectrometry, ^1^H NMR, ESR, XRD, and thermal studies. By these spectral studies it is found that Co(II), Ni(II), and Cu(II) complexes have exhibited octahedral geometry whereas the Zn(II), Cd(II), and Hg(II) complexes have exhibited tetrahedral geometry. Potentiometric studies have been carried out on complexes of Schiff base (BHMQMHI) with Cu(II), Co(II), and Ni(II). Calvin-Bjerrum pH-titration technique as used by Irving and Rossotti has been applied to determine stability constants in mixed solvents at 25 ± 1°C. The present study reports the protonation constants of this ligand and stability constants of its metal complexes in dioxane-water (50%, v/v) mixtures. Metal-ligand stability constants fall in the order of Cu(II) > Co(II) > Ni(II) which is in agreement with those reported by Irving stability order. The Schiff base (BHMQMHI) and its metal complexes have been screened for their *in vitro* antibacterial and antifungal activities by minimum inhibitory concentration (MIC) method. The DNA cleavage activities of all the complexes were studied by agarose gel electrophoresis method. In addition, the free ligand along with its complexes has been studied for their antioxidant activity.

## 1. Introduction

Schiff base complexes have undergone a phenomenal growth during the recent years because of the versatility offered by these complexes in the field of industries, catalysis and biological system, and so forth. In this way, the synthesis, structural investigation, and reaction of transition metal Schiff bases have received a special attention, because of their biological activities as antitumoral, antifungal, and antiviral activities [1].

Hydrazones possessing an azomethine proton (–NHN=CH–) constitute an important class of compounds for new drug development. Many researchers have synthesised these compounds as well as their metal complexes as target structures and evaluated their biological activities. These observations have guided the development of new hydrazones with varied biological activities. The biological activity of complexes derived from hydrazones has been studied and contrasted with regard to their antibacterial, antitumoral, antiviral, antimalarial, and antitubercular properties. It has also been shown that the azomethine N, which has a lone pair of electrons in an *sp* ^2^ hybridised orbital, is biologically important [2]. The isatin molecule (1H-indole-2, 3-Dione) is a versatile moiety that displays diverse biological activities. In this paper, we report the synthesis, structural characterization, thermal decomposition, and biological studies of some transition metal complexes with the host of a bishydrazone derived from 5-bromo-3-hydrazonoindolin-2-one and 7-formyl-8-hydroxy-2-methylquinoline,* namely*, (5-bromo-3-(((8-hydroxy-2-methylquinolin-7-yl)methylene)hydrazono)indolin-2-one (BHMQMHI)).

## 2. Experimental

### 2.1. Materials and Reagents

All chemicals used were of analytical grade. 5-Bromo isatin and hydrazine hydrate (99%) were obtained from Fluka and Sisco chemicals.

### 2.2. Solutions

Dioxane was purified by the standard procedures [3]. Ligand solution was prepared in double distilled alcohol. Metal salt solutions were prepared by dissolving the corresponding metal salt in double distilled deionized water and standardized by standard volumetric methods [3].

*Calvin-Bjerrum Technique.* The experimental procedure involves the acid titration, ligand titration, and metal titration. The details of titrations are shown in Table 1. The total volume in all the cases was 50 mL.

### 2.3. Physical Measurements

pH measurements were carried out using Elico pH-meter model L1-122. Elemental analysis was carried out using a Heracus Carlo Erba 1108 CHN analyzer at STIC, Cochin. The IR spectra of the Schiff base (BHMQMHI) and its Co(II), Ni(II), Cu(II), Zn(II), Cd(II), and Hg(II) complexes were recorded in the region of 4000–250 cm^−1^ on a Perkin Elmer Spectrum RX-IFTIR spectrophotometer. The electronic spectra of the Co(II), Ni(II), and Cu(II) complexes were recorded on an ELICO SL-164 double beam UV-visible spectrophotometer in the range of 200–1200 nm in DMF (10^−3^ M) solution. Magnetic susceptibility measurements were made at room temperature on a Gouy balance using Hg[Co(NCS)_4_] as the calibrant. Molar conductivity measurements were recorded on an ELICO CM-180 conductivity bridge in DMF solution (10^−3^ M) using a dip-type conductivity cell fitted with a platinum electrode. The ^1^H NMR spectra were recorded in DMSO-d_6_ on a Bruker 500 MHz spectrophotometer using TMS as an internal standard. The mass spectra were recorded on a JEOL GC mate mass spectrophotometer. The ESR spectrum of the Cu(II) complex in the polycrystalline state was recorded on a Varian-E-4X band EPR spectrophotometer using TCNE as the “*g*” marker (*g* = 2.00277) at room temperature. Thermal analyses were measured from room temperature to 1000°C in N_2_ on a perkin Elmer, Diamond TG/DTA model thermal analyzer at STIC, Cochin, with a heating rate of 10°C min^−1^. The XRD patterns of the Cu(II) complex were recorded on a Bruker AXS D8 Advance X-ray diffractometer using Cu K*α* = 1.5404 radiation (*λ* Å).

### 2.4. Procedures

#### 2.4.1. Potentiometric Measurements

The potentiometric measurements were carried out at 25 ± 1°C and at constant ionic strengths (0.1 M and 0.2 M). The pH-meter was calibrated before each titration using standard buffers. The ionization constants of the investigated Schiff base (BHMQMHI) and the stability constants of its metal complexes with Co(II), Ni(II), and Cu(II) ions were determined potentiometrically using the technique of Calvin-Bjerrum Technique. The ionization constants of the ligand are calculated using the equation used by Irving and Rossotti while metal-ligand stability constants were calculated using methods applied for computing successive stability constants.

#### 2.4.2. Synthesis of Schiff Base Ligand

**Figure 1:**
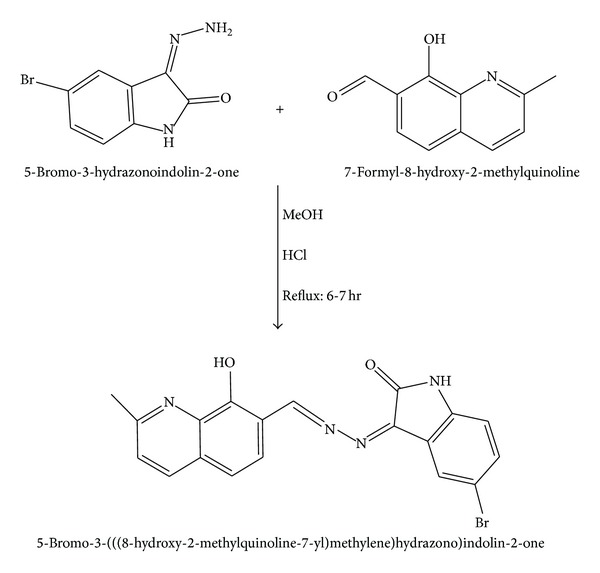
Schematic representation of Schiff base (BHMQMHI).

*5-Bromo-3-(((8-hydroxy-2-methylquinolin-7-yl)methylene)hydrazono)indolin-2-one (BHMQMHI):* the Schiff base has been synthesized by refluxing the equimolar mixture of hot methanolic solution of 7-formyl-8-hydroxy-2-methylquinoline (0.01 mol, 30 mL) and hot methanolic solution of 5-bromo-3-hydrazonoindolin-2-one (0.01 mol, 30 mL) for 6-7 h in presence of catalytical amount of hydrochloric acid. The product obtained after the evaporation of the solvent was filtered, washed with cold methanol, and finally recrystallized from methanol to afford Schiff base (BHMQMHI) as shown in Figure 1. The purity of the compound has been checked by thin layer chromatography (TLC).

#### 2.4.3. Synthesis of Metal Complexes

The metal complexes were prepared using metal chlorides and the Schiff base (BHMQMHI) by the general method. An ethanolic solution (40 mL) of Schiff base and Co(II), Ni(II), and Cu(II) chlorides in 1 : 2 molar ratio and 1 : 1 molar ratio for Zn(II), Cd(II), and Hg(II) complexes were refluxed on water bath for about 4 h. An aqueous solution of sodium acetate was added to the reaction mixture to adjust the pH to 6.0-7.0 and reflux was further continued for about an hour. The separated solid complexes were filtered off and washed thoroughly with water and then with little warm ethanol. The complexes obtained were finally dried under vacuum desiccator over fused CaCl_2_. Elemental analysis data are shown in Table 2. The complexes were analyzed for their metal and chloride contents by standard methods [3].

## 3. Pharmacology

### 3.1. Antibacterial and Antifungal Activities

Antibacterial and antifungal activity of ligand and their complexes has been determined by serial tube dilution technique [4]. The stock solution of each compound was prepared by dissolving 10 mg of each test compound in 10 mL of freshly distilled DMSO. The various concentrations of the test compounds (100, 75, 50, 25, and 12.5 *μ*g/mL^−1^) were prepared by diluting the stock solution with the required volume of freshly distilled DMSO. The MIC was measured after 24 h in case of antibacterial activity and 48 h for antifungal activity.

The* in vitro* biological screening effects of the investigated compounds were tested against the bacteria* S. aureus*,* B. subtilis*, and* P. aeruginosa* cultured on Mueller Hinton agar media. The antifungal activity of the Schiff base and its metal complexes was tested against* A. flavus*,* A. niger*, and* C. albicans* on Czapek-Dox agar media. The control containing only DMSO and the standard antibiotic (Gentamycin and Amphotericin) was also kept for comparison [5].

### 3.2. Antioxidant Activity: Radical Scavenging Activity

The radical scavenging activity ligand (BHMQMHI) and its complexes were determined by using 1, 1-diphenyl-2, 2-picryl hydrazyl-free radical (DPPH) assay method [6]. DPPH is a stable free-radical molecule, accept an electrons or hydrogen radicals to become a stable diamagnetic molecule and also contained an odd electron in its structure that is frequently used for detection of the radical scavenging activity in chemical analysis. The reduction capability of DPPH radicals was determined by a decrease in its absorbance at 517 nm induced by antioxidants. The absorption maximum of a stable DPPH radical in ethanol was at 517 nm. The absorbance decreased when the DPPH is scavenged by an antioxidant, through donation of hydrogen to form a stable DPPH molecule. In the radical form, those molecules show an absorbance at 517 nm, which disappeared after acceptance of an electron or hydrogen radical from an antioxidant compound to become a stable diamagnetic spin-paired molecule. The stock solutions (1 mg/mL) of the ligand and its complexes were diluted to a final concentration of 25, 50, 75, and 100 *μ*g/mL in methanol. DPPH solution (1 mL, 0.1 mmol) was added to 2.5 mL of test solution of different concentration and allowed to react at room temperature. After 30 min the absorbance was measured at 517 nm. A graph was plotted with percentage scavenging effects on the *y*-axis and concentration (*μ*g/mL) on the *x*-axis. Radical scavenging activity was expressed as a percentage and was calculated using the following formula:

(1) Scavenging  effect(%)=Control  OD−Sample  ODControl  OD×100.

The scavenging capability of the ligand and its complexes were compared with standard drugs, namely, butylated hydroxyl anisole (BHA) and tertiary butylated hydroxyl quinoline (TBHQ) and ascorbic acid.

### 3.3. DNA Cleavage Experiment

Preparation of culture media and DNA isolation of Calf-thymus were done according to the literature procedure [7]. Nutrient broth (10 g L^−1^ of peptone, 5 g L^−1^ of yeast extract and 10 g L^−1^ of NaCl) was used for culturing of Calf-thymus.

The cleavage of Calf-thymus DNA was determined by agarose gel electrophoresis [5]. Calf-thymus DNA was cultured, isolated, and used as DNA for the experiment. The precipitated DNA was separated by centrifugation and the pellet was dried and dissolved in tris buffer (10 mM tris pH 8.0) and stored in cold condition. 25 *μ*g of the test samples was added to the isolated Calf-thymus DNA and incubated for 2 h at 37°C. After incubation, the samples were electrophoresed for 45 min at 50 V on agarose gel using TAE buffer (4.84 g tris base, pH 8.0, 0.5 M EDTA/L). After the run, gel was removed and stained with 10 *μ*g/mL ethidium bromide (ETBR) for 10–15 min and the image was taken in UV transilluminator and photographed to determine the extent of DNA cleavage. The results are compared with standard DNA marker [8–10].

## 4. Results and Discussion

The Schiff base ligand and its transition metal complexes have been synthesized and characterized by spectral and elemental analytical data. All the complexes are freely soluble in acetonitrile, DMF, and DMSO and insoluble in water. The analytical data (Table 2) indicates that the stoichiometry of the complexes is 1 : 2 (metal to ligand ratio) for Co(II), Ni(II), Cu(II), and 1 : 1 (metal to ligand ratio) for Zn(II), Cd(II), and Hg(II) complexes. The lower conductance values (18–29 Ohm^−1^ cm^2^ mol^−1^) of the complexes support their nonelectrolytic nature of the compounds [11, 12].

### 4.1. IR Spectral Studies

The prominent infrared spectral data with the tentative assignments of the Schiff base (BHMQMHI) and its Co(II), Ni(II), Cu(II), Zn(II), Cd(II), and Hg(II) complexes are presented in Table 3.

The broad peak observed at 3364 cm^−1^ in the IR spectra of the ligand assigned to *ν*(OH), which was found to have disappeared in all the complexes, thereby indicating deprotonation and formation of metal-oxygen bond [13]. This is further supported by the shifting of phenolic *ν*(C–O) towards higher frequency, indicating the coordination of the phenolate oxygen to metal ion. The *ν*(HC=N) vibration of the ligand occurs at 1583 cm^−1^, which is shifted to a lower frequency in the complexes, indicating the involvement of the azomethine nitrogen in chelation with the metal ion [14]. The band corresponding to *ν*(C=O) at 1696 cm^−1^ is shifted to a lower frequency, supporting the coordination of the carbonyl oxygen [15]. However vibrational characteristics of the ring *ν*(N–H) and *ν*(C=N) of the ketimine moiety remain almost unaffected, indicating the nonparticipation of these groups in coordination [16]. The bands in the regions 500–598 and 468–496 cm^−1^ are ascribed to *ν*(M–O) and *ν*(M–N) vibrations, respectively [17]. The bands observed in the region 344–366 cm^−1^ are due to the formation of *ν*(M–Cl) bond, which was characteristic of the involvement of chloride atom in coordination with Zn(II), Cd(II) and Hg(II) ions. But in Co(II), Ni(II), and Cu(II) complexes *ν*(M–Cl) bands are absent. From the above observations, it can be concluded that the ligand binds to the metal ion in a tridentate fashion through the deprotonated phenolate oxygen, Schiff base nitrogen, and the carbonyl oxygen of the isatin moiety.

### 4.2. ^1^H-NMR Spectral Studies

^1^H-NMR spectra of the ligand (BHMQMHI) and its Zn(II), Cd(II), and Hg(II) complexes were recorded in DMSO-d^6^ using TMS as an internal standard and the integrated intensities of the signals are well agreed with the number of protons of each type. The signal at *δ* (10.3) (s, 1H) was assigned to phenolic −OH in the ligand. The signal is disappear in Zn(II), Cd(II) and Hg(II) complexes, which indicates the involvement of phenolic oxygen atom in the coordination* via* deprotonation [18]. The signal at *δ* (8.4) (s, 1H) is due to the azomethine group in the ligand (BHMQMHI). These signals shifts downfield in the regions *δ* (9.1) (s, 1H), *δ* (8.8) (s, 1H), and *δ* (9.0) (s, 1H) in the spectra of Zn(II), Cd(II), and Hg(II) complexes, respectively, and confirms the coordination of “N” of (–C=N–) group in bonding with the metal ions [19]. The peak that appeared at *δ* (9.7) (s, 1H) is due to the hydrogen of –NH in the ligand, but in case of Zn(II), Cd(II), and Hg(II) complexes the peak was observed at *δ* (9.7) (s, 1H), and this signal remains unaltered in the spectra of Zn(II), Cd(II), and Hg(II) complexes confirming the noncoordination (–NH) in bonding with metal ions [20]. In ligand seven aromatic protons have been observed in the region *δ* (6.9–7.5) (m, 7H) as a multiplet, and it is shifted downfield in the Zn(II), Cd(II), and Hg(II) complexes. The signals observed at *δ* (2.5) are due to methyl protons that remain almost unaffected in Zn(II), Cd(II), and Hg(II) complexes indicating the nonparticipation of these groups in coordination [21].

### 4.3. Mass Spectral Studies

The mass spectrum of the Schiff base (BHMQMHI) shows a molecular ion peak at* m/z* 409.24, which is equivalent to its molecular weight. The mass spectra of the Co(II), Ni(II), Cu(II), Zn(II), Cd(II), and Hg(II) complexes showed a molecular ion peak at* m/z* 875.39, 875.15, 880.00, 509.09, 556.09, and 644.27 which is the same as that of the molecular weight of the complexes. This confirms the proposed structure for the complexes.

### 4.4. Electronic Spectral and Magnetic Studies

The electronic spectral data of metal (II) complexes were recorded in DMF as shown in Table 4. They have been studied with the view to obtain more information on stereochemistry of the complexes and to procedure more support for the conclusion, deduced with the help of magnetic data. The Co(II) complex of the electronic absorption bands appears at 15384 and 20000 cm^−1^ due to ^4^T_1g_ (F) →^4^A_2g_ (F) (*ν*_2_) and ^4^T_1g_ (F) →^4^T_2g_ (P) (*ν*_3_) transitions, respectively, in an octahedral environment [22]. The band *ν*_1_ could not be observed because of its very low intensity. However the position of the *ν*_1_ band has been computed (7148 cm^−1^) by the equation *ν*_1_ = *ν*_2_ − 10 Dq. The ligand field parameters such as Dq,* B*′, *β*, and *β*% have been calculated by using band-fitting equation given by Underhill and Billing [23]. The crystal field splitting (Dq) energy value was at 823 cm^−1^. These values are well within the range and also reported by most of the octahedral Co(II) complexes. Co(II) complex under present investigation process interelectronic repulsion parameter (*B*′) 929 cm^−1^. The Racah parameter is less than free ion value suggesting a considerable orbital overlap and delocalization of electrons on the metal ion. The nephelauxetic ratio (*β*) for the Co(II) complex is 0.89. This is less than one, suggesting partial covalency in the metal ligand bond. The values Dq, *β*, *β*%, *ν*_2_/*ν*_1_, and LFSE suggest the octahedral geometry for Co(II) complex [24]. The Co(II) complex displays a magnetic moment value of 4.88 BM, which is within the range of 4.46–5.53 BM.

The electronic spectrum of Ni(II) complex shows two bands at 15384 and 25000 cm^−1^ assignable to ^3^A_2g_ (F) →^3^T_1g_ (F) (*ν*_2_) and ^3^A_2g_ (F) →^3^T_1g_ (P) (*ν*_3_) transitions, respectively, in an octahedral environment [25]. The lowest band *ν*_1_ was not observed due to limited range of the instrument used. However, it is calculated by using equation suggested by Billing and Underhill. Racah parameter* B*′ is less than the free ion value of 1040 cm^−1^ indicating the covalent character of the complex. The ratios *ν*_2_/*ν*_1_ and *β*% further support the octahedral geometry around the Ni(II) ion [26]. The Ni(II) complex showed the magnetic moment value of 3.00 BM, which is within the range of 2.7–3.3 BM.

The Cu(II) complex exhibits a broad asymmetric band in the region 14285–17391 cm^−1^. The broadness of the band may be due to dynamic Jahn-Teller distortion and is assigned to ^2^B_1g_→^2^A_2g_ (*ν*_1_), ^2^B_1g_→^2^E_g_ (*ν*_2_), and ^2^B_1g_→^2^B_2g_ (*ν*_3_) transitions. The Cu(II) complexes showed magnetic moment value of 1.94 BM, which is within the range of 1.75–2.20 BM which is consistent with octahedral geometry [27].

### 4.5. Thermogravimetric Analysis

The Copper (II) complex is subjected to thermogravimetric analysis in dynamic air in 40–750°C temperature range, at a heat ingrate of 10°C/min. The complex is stable up to 200°C and exhibits a single stage decomposition pattern, as is evident from the TG-DTA profile. The mass loss in the single stage decomposition occurred at 221.00°C which can be attributed to the loss of ligand moiety further leaving behind the metal oxide residue. The complex shows gradual degradation up to 709.3°C. The single stage decomposition of the metal complex usually occurs when there is a high degree of electron delocalization along a conjugated system which leads to uniformity in bond strength [28].

### 4.6. ESR Spectrum of Cu(II) Complex

The ESR spectrum of the Cu(II) complex in a polycrystalline state was recorded at room temperature. The *g*_||_ and *g*_⊥_ values were found to be 2.405 and 2.041, respectively. The *g*_*av*_ was calculated to be 2.057. The broadening of this signal might be due to dipolar interactions, indicating lowered site symmetry suggesting that the unpaired electron resides mainly in the *d*_*x*2-d*y*2_ orbital [29]. The axial symmetry parameter “*G*” was determined as *G* = (*g*_||_ − 2.00277)/(*g*_⊥_ − 2.00277) = 4.455, suggesting that there is no exchange interaction in the Cu(II) complex [30].

### 4.7. Powder X-Ray Diffraction

**Figure 2:**
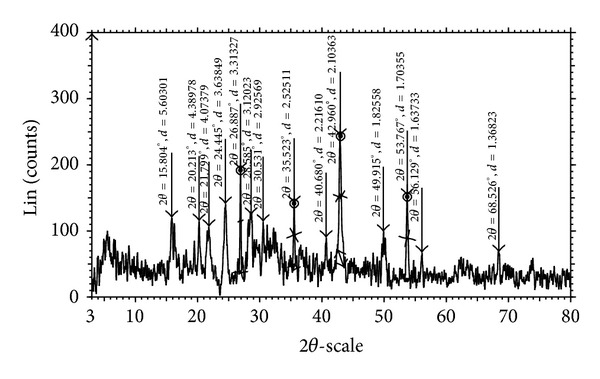
Powder XRD pattern of Cu(II) complex.

The Cu(II) complex has been characterized by powder XRD studies with a view to find the type of crystal system. The XRD data of Cu(II) complex is given in Table 5. There are 11 reflections (2*θ*) between 15.804 and 68.526 with maxima at 2*θ* = 35.523 corresponding to the value of *d* = 2.525. The interplanar spacing (*d*) has been calculated from the positions of intense peaks using Bragg's equation *nλ* = 2*dSin*⁡*θ* (where *λ* = 1.54056 Å). The observed and calculated values of *d* are quite consistent. The experimental values of sin^2^ *θ*/common factor are recorded for each peak in Figure 2. The *h* ^2^ + *k* ^2^ + *l* ^2^ values of the complex were found to be 1, 2, 3, 4, 5, 6, 7, 9, 11, 12, and 17. The presence of forbidden number 7 indicates the Cu(II) complex belongs to hexagonal system [31].

### 4.8. Potentiometric Determination of the Ionization Constants

**Figure 3:**
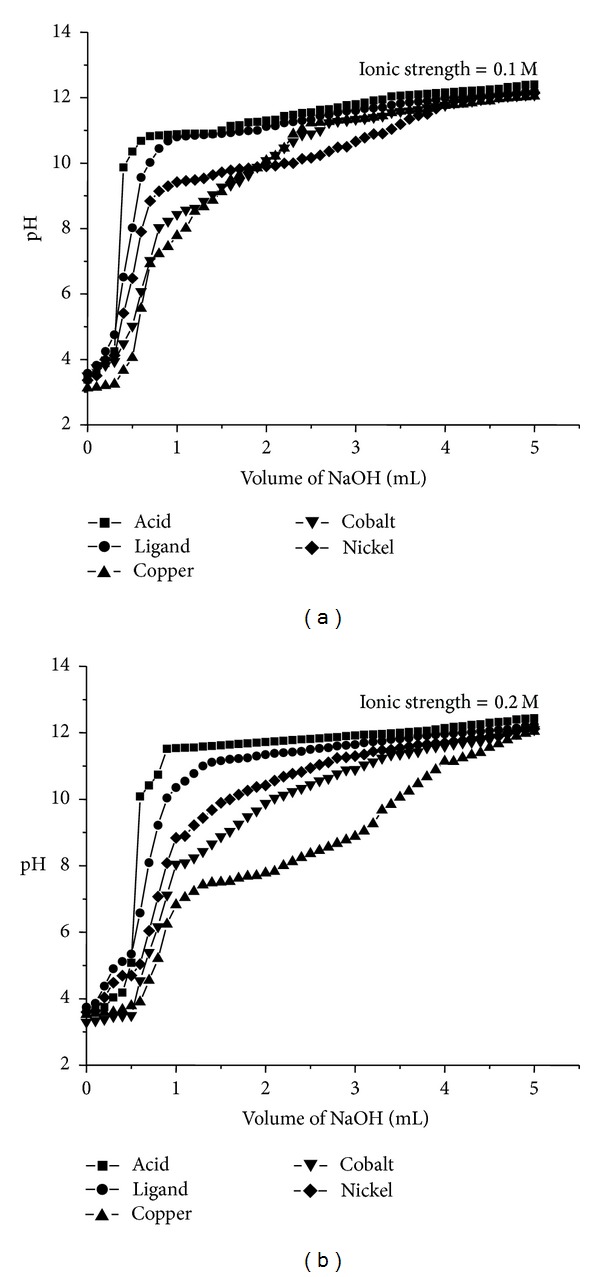
Plot of pH* versus* volume of NaOH added.

The ionization constants of the ionizable group in Schiff base under investigation are determined by a method similar to that described by Calvin-Bjerrum [32]. The average of protons associated with the ligand $\left({\overset{-}{n}}_{A}\right)$ at different pH values is calculated utilizing acid and ligand titration curves (Figures 3(a) and 3(b)). The pKa values can be calculated from the curves obtained by plotting ${\overset{-}{n}}_{A}$ versus pH. The formation curves are found between 0 and 1. This indicates that the ligands have one dissociable proton. Ligand exhibits only one pKa value in the range of 10.0 in 0.1 M ionic strength and 9.4 in 0.2 M ionic strengths, respectively, in 50% dioxane-water, this can be attributed to the ionization of phenolic –OH of the Schiff base (BHMQMHI).

### 4.9. Potentiometric Determination of the Stability Constants

The stability constants of the Co(II), Ni(II), and Cu(II) complexes with (BHMQMHI) are determined potentiometrically using the method described by Calvin-Bjerrum. The formation curves of the investigated complexes are obtained by plotting a graph between the average number of ligands attached per metal ion $\left(\overset{-}{n}\right)$ and the free ligand exponent (pL). Values of $\overset{-}{n}$ and pL are calculated. The maximum $\overset{-}{n}$ values calculated for metal-ligand system are found not to exceed two indicating the formation of 1 : 1 and 1 : 2 (metal : ligand) complexes. The mean log⁡*K* and log⁡*β* values of the Co(II), Ni(II), and Cu(II) complexes with Schiff base (BHMQMHI) are listed in Table 6. The order of stability constants is found to be Cu(II) > Co(II) > Ni(II) in accordance with the Irving and Williams order [33] for divalent metal ions of the 3d series. It is clear from Table 6 that the stability of Cu(II) complexes is considerably larger as compared to other metals of the 3d series. Under the influence of the ligand field, Cu(II) (3*d* ^9^) will receive some extra stabilization [34] due to tetragonal distortion of octahedral symmetry in their complexes. The Cu(II) complexes will be further stabilized due to the Jahn-Teller effect [35].

## 5. Pharmacology Results

### 5.1. Antimicrobial Evaluation of Ligand (BHMQMHI) and Its Metal (II) Complexes

The ligand and metal complexes were screened for antibacterial activity and the results are presented in Table 7. The synthesized Schiff base (BHMQMHI) has an inhibitory effect (MIC values of 75–100 *μ*g/mL^−1^) on growth of the tested bacterial strains. All complexes showed greater bactericidal activities against* S. aureus* (MIC 12.5–50 *μ*g/mL^−1^),* B. subtilis* (MIC 12.5–50 *μ*g/mL^−1^), and* P. aeruginosa* (MIC 12.5–50 *μ*g/mL^−1^) than the ligand. In the fungal studies, the ligand had an inhibitory effect (MIC values in range 50–100 *μ*g/mL^−1^) on the growth of the tested strains and complexes again showed greater fungicidal activities against* A. flavus* (MIC 12.5–75 *μ*g/mL^−1^),* A. niger* (MIC 12.5–25 *μ*g/mL^−1^), and* C. albicans* (MIC 12.5–50 *μ*g/mL^−1^). Co(II) and Zn(II) complexes had greater bacterial and fungal activities than the ligand (BHMQMHI).

All the metal complexes individually exhibited varying degrees of inhibitory effect on the growth of the tested bacterial/fungal species. Table 7 shows the activity of the metal complexes became more pronounced when coordinated with the metal ions [36]. This enhancement in the activity may be due to the structure of Schiff base ligand by possessing an azomethine (C=N) linkage. The toxic activity of the complexes with the ligand can be ascribed to the increase in the lipophilic nature of the complexes arising from chelation. The mode of action of complexes involves the formation of hydrogen bonds with the imino group by the active sites leading to interference with the cell wall synthesis. This hydrogen bond formation damages the cytoplasmic membrane and the cell permeability may also be altered leading to cell death [37].

A comparative study of the ligand and complexes (MIC values) indicated that the complexes exhibited higher antimicrobial activity than the free ligand. Such increased activity of the complexes can be explained on the basis of Overtone's concept and Tweedy's chelation theory [38]. These complexes also disturb the respiration process of the cell and thus block the synthesis of the proteins, restricting the further growth of the organism. Furthermore, the mode of action of the compound may involve the formation of a hydrogen bond through the azomethine group with the active center of the cell, resulting in interference with the normal cell processes. In general, metal complexes are more active than ligands because metal complexes may serve as a vehicle for the activation of ligands as the principle cytotoxic species [39].

### 5.2. Antioxidant Activity: Radical Scavenging Activity

**Figure 4:**
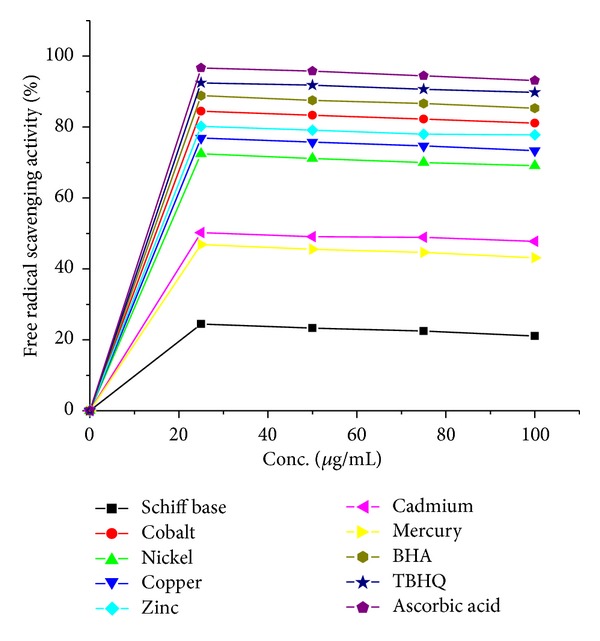
Antioxidant results of Schiff base and its metal (II) complexes.

The metal (II) complexes prepared from Schiff base (BHMQMHI) were subjected for free radical scavenging activity by DPPH method [5, 6]. From the investigation it was clearly observed that metal complexes scavenge DPPH effectively than Schiff base (BHMQMHI). The Co(II), Zn(II), Cu(II), and Ni(II) complexes show superior activity while Cd(II) and Hg(II) complexes are moderate activity as a radical scavenger compound with standards as shown in Figure 4.

### 5.3. DNA Cleavage Efficiency

**Figure 5:**
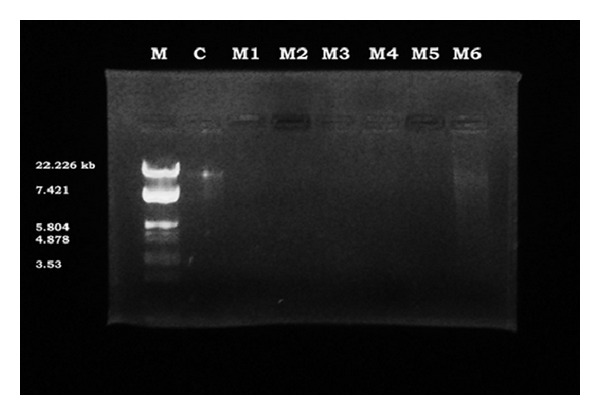
Gel picture showing the cleavage analysis of samples.

The Schiff base metal complexes were subjected to their DNA cleavage activity by agarose gel electrophoresis method [5]. From Figure 5, it was clearly indicated that the Co(II) (lane M1), Ni(II) (lane M2), Cu(II) (lane M3), Zn(II) (lane M4), and Cd(II) (lane M5) shows complete DNA cleavage activity and Hg(II) (lane M6) complex shows partial DNA cleavage activity. The Schiff base metal complexes have acted on DNA effectively, since as there was molecular weight difference between the control and the treated DNA samples. The completion of gel electrophoresis experiment clearly indicated that the intensity of the treated DNA samples has diminished due to the cleavage of DNA. These results indicated that the metal ions played an important role in the cleavage of DNA [40]. The information obtained in this study could be helpful in the understanding of the mechanism of interactions of metal (II) complexes with nucleic acids and should be useful in the development of potential probes for investigation of the structure and conformation of DNA or new therapeutic agents for some diseases [41].

## 6. Conclusion

**Figure 6:**
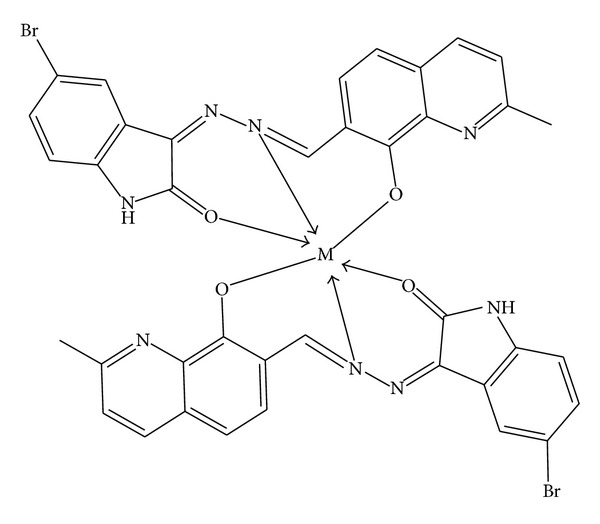
Proposed structure of Co(II), Ni(II), and Cu(II) metal complexes (octahedral).

**Figure 7:**
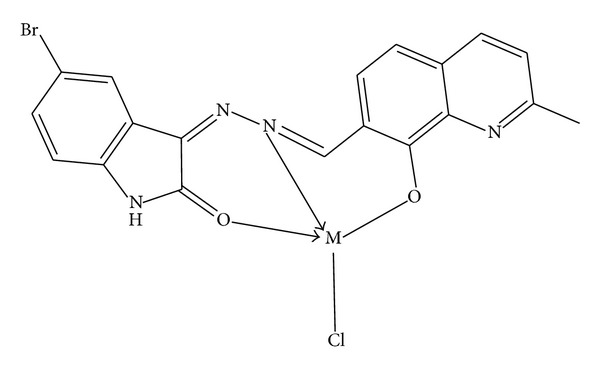
Proposed structure of Zn(II), Cd(II), and Hg(II) metal complexes (tetrahedral).

This study shows that synthesized Schiff base (BHMQMHI) acts as tridentate ligand coordinating to metal ion through azomethine nitrogen, carbonyl oxygen, and phenolic oxygen atom* via* deprotonation. The synthesized new Schiff base and its metal complexes have been confirmed by the analytical data, IR, electronic, mass spectrometry, ^1^H NMR, ESR spectral data, magnetic susceptibility, molar conductance, XRD, and thermal studies. The results of the potentiometric studies on complexes of Schiff base (BHMQMHI) with Cu(II), Co(II), and Ni(II) using Calvin-Bjerrum pH-titration technique as used by Irving and Rossotti indicate that the order of stability is Cu(II) > Co(II) > Ni(II). This order is in accordance with Irving-Williams order of stability and formation constants log⁡*K* and log⁡*β* values are decreases as the ionic strength increases. This observation is in agreement with Debye-Hukel equation. The synthesized Schiff base metal complexes show better antibacterial and antifungal activity than the ligand. The Co(II), Ni(II), Cu(II), Zn(II), Cd(II), and Hg(II) complexes were also found to show significant antioxidant and DNA cleavage activity. Based on the analytical and spectral studies, we propose octahedral geometry for the Co(II), Ni(II), and Cu(II) complexes and tetrahedral geometry for the Zn(II), Cd(II), and Hg(II) complexes. (Figures 6 and 7).

## Figures and Tables

**Table 1:** The experimental procedure involves the acid titration, ligand titration, and metal titration. *T* = 25 ± 1°C  50% Dioxane-water  *µ* = 0.1 M/0.2 M NaClO_4_.

Solution (initial concentration)	Acid titration	Ligand titration	Metal titration
HClO_4_ (0.01 M)	5.0 mL	5.0 mL	5.0 mL
NaClO_4_ (1 M/2 M)	5.0 mL	5.0 mL	5.0 mL
Dioxane	25.0 mL	25.0 mL	25.0 mL
Water	15.0 mL	10.0 mL	5.0 mL
Ligand (0.01 M)	—	5.0 mL	5.0 mL
Metal (0.01 M)	—	—	5.0 mL

**Table 2:** Physical and analytical data of the ligand (BHMQMHI) and its metal (II) complexes.

Compound	Molecular formula	Yield (%)	Found (calculated) %					*Ω*m (*Ω* ^−1^ cm^2^ mol^−1^)	*µ*_eff_ (BM)
			C	H	N	M	Cl		
Ligand	[C_19_H_13_N_4_O_2_Br]	81	55.76 (55.54)	3.20 (3.16)	13.69 (13.53)	—	—	—	—
Co(II) complex	[Co(C_38_H_24_N_8_O_4_Br_2_)]	79	52.14 (52.01)	2.76 (2.68)	12.80 (12.78)	6.73 (6.68)	—	27.23	4.88
Ni(II) complex	[Ni(C_38_H_24_N_8_O_4_Br_2_)]	70	52.15 (52.08)	2.76 (2.63)	12.80 (12.76)	6.70 (6.63)	—	20.43	3.00
Cu(II) complex	[Cu(C_38_H_24_N_8_O_4_Br_2_)]	65	51.86 (51.78)	2.75 (2.68)	12.73 (12.63)	7.22 (7.12)	—	18.45	1.94
Zn(II) complex	[Zn(C_19_H_12_N_4_O_2_Br)Cl]	80	44.83 (44.77)	2.38 (2.28)	11.01 (10.08)	12.84 (12.70)	6.96 (6.76)	22.81	—
Cd(II) complex	[Cd(C_19_H_12_N_4_O_2_Br)Cl]	77	41.04 (39.93)	2.18 (2.00)	10.08 (9.93)	20.21 (20.19)	6.37 (6.26)	23.76	—
Hg(II) complex	[Hg(C_19_H_12_N_4_O_2_Br)Cl]	66	35.42 (34.54)	1.88 (1.78)	8.70 (8.65)	—	—	29.76	—

**Table 3:** IR spectral bands of the ligand (BHMQMHI) and its metal complexes (cm^−1^).

Tentative assignments	L	Co(II) complex	Ni(II) complex	Cu(II) complex	Zn(II) complex	Cd(II) complex	Hg(II) complex
Hydrogen bonded OH group	3364	—	—	—	—	—	—
Indole ring NH	3203	3203	3203	3203	3023	3203	3203
**ν**(C=O) ring	1696	1685	1686	1679	1647	1672	1667
**ν**(C=N) ring	1603	1603	1603	1603	1603	1603	1603
**ν**(C=N) aldimine	1583	1565	1525	1530	1533	1505	1549
**ν**(C–O)	1289	1300	1307	1306	1314	1344	1360
**ν**(N–N)	991	1067	1082	1035	1099	1059	1033
**ν**(M–N)	—	479	496	468	477	489	489
**ν**(M–O)	—	533	578	500	500	523	598
**ν**(M–Cl)	—	—	—	—	344	358	366

**Table 4:** Electronic spectral bands and ligand field parameters of the Co(II), Ni(II), and Cu(II) complexes in DMF (10^−3^ M) solution.

Complexes	Transitions in cm^−1^			Dq (cm^−1^)	*B*′ (cm^−1^)	*β*	*β*%	*ν*_2_/*ν*_1_	LFSE (k cal)
	*ν*_1_	*ν*_2_	*ν*_3_						
Co(II) complex	7148	15384	20000	823	929	0.89	10.64	2.15	14.11
Ni(II) complex	9614	15384	25000	961	769	0.73	26.02	1.60	32.96
Cu(II) complex		14285–17391		1583	—	—	—	—	27.15

**Table 5:** X-ray diffraction data of Cu(II) complex.

2*θ*	*θ*	sin⁡⁡*θ*	sin⁡^2^⁡*θ*	*h* ^2^ + *k* ^2^ + *l* ^2^	*h k l *	*d* value		*a* in Å
						Cal	Abs	
15.804	7.902	0.1374	0.0189	1	1 0 0	5.6030	5.6008	5.600
24.445	12.222	0.2117	0.0448	2.37234 (2)	1 1 0	3.6384	3.6363	5.600
26.887	13.444	0.2324	0.0540	2.85970 (3)	1 1 1	3.3132	3.3120	5.600
30.581	15.290	0.2637	0.0695	3.67509 (4)	2 2 0	2.9256	2.9198	5.600
35.523	17.761	0.3050	0.0930	4.92361 (5)	2 1 0	2.5251	2.5241	5.600
40.680	20.340	0.3475	0.1208	6.39237 (6)	2 1 1	2.2161	2.2152	5.600
42.960	21.480	0.3661	0.1340	7.09426 (7)	—	2.1036	2.1028	5.600
49.915	24.957	0.4219	0.1780	9.41976 (9)	2 2 1	1.8255	1.8248	5.600
53.767	26.883	0.4521	0.2044	10.8179 (11)	3 1 1	1.7035	1.7028	5.600
56.129	28.064	0.4704	0.2213	11.7106 (12)	2 2 2	1.6373	1.6366	5.600
68.526	34.263	0.5629	0.3169	16.7699 (17)	3 2 2	1.3682	1.3676	5.600

**Table 6:** Cumulative data of log⁡⁡k and log⁡⁡*β* values for Schiff base (BHMQMHI) complexes.

Solvent	Ionic strength = 0.1 M						Ionic strength = 0.2 M					
	pKa						pKa					
	10.0						9.4					
	Copper		Cobalt		Nickel		Copper		Cobalt		Nickel	
	log⁡⁡*k*	log⁡⁡*β*	log⁡⁡*k*	log⁡⁡*β*	log⁡⁡*k*	log⁡⁡*β*	log⁡⁡*k*	log⁡⁡*β*	log⁡⁡*k*	log⁡⁡*β*	log⁡⁡*k*	log⁡⁡*β*
50% Dioxane-water	5.644	3.900	5.554	5.025	4.5199	4.2300	5.499	5.0325	5.476	3.536	4.442	3.835

**Table 7:** The antimicrobial activity of ligand and its metal (II) complexes evaluated by MIC (*μ*g/mL^−1^).

Schiff base/complexes	*S. aureus *	*B. subtilis *	*P. aeruginosa *	*A. flavus *	*A. niger *	*C*. *albicans *
Ligand	100	75	75	100	50	75
Co(II) complex	12.50	12.50	12.50	12.50	12.50	12.50
Ni(II) complex	12.50	25	25	25	25	12.50
Cu(II) complex	25	50	12.50	75	12.50	25
Zn(II) complex	12.50	12.50	12.50	12.50	12.50	12.50
Cd(II) complex	50	50	25	50	25	50
Hg(II) complex	25	12.50	50	12.50	25	12.50
Gentamycine	12.50	12.50	12.50	—	—	—
Amphotericin	—	—	—	12.50	12.50	12.50
